# A meta-analysis of home range studies in the context of trophic levels: Implications for policy-based conservation

**DOI:** 10.1371/journal.pone.0173361

**Published:** 2017-03-07

**Authors:** Catherine Fauvelle, Rianne Diepstraten, Tyler Jessen

**Affiliations:** 1 Faculty of Environmental Design, University of Calgary, Calgary, Canada; 2 Faculty of Veterinary Medicine, University of Calgary, Calgary, Canada; Sveriges lantbruksuniversitet, SWEDEN

## Abstract

Home ranges have been widely-used as ecological tools, though using home range estimates in decision-support for conservation biology is a relatively new idea. However, trophic levels are rarely taken into consideration when estimating home range. This lapse could present issues when interpreting past studies, especially in policy-based conservation. The objectives of this study were to survey the current literature, to critically analyse published articles with home range analyses, and to compare home range size by species’ trophic level. We predicted that animals residing in higher trophic levels would have significantly larger home ranges than animals occupying lower trophic levels. We found that terrestrial carnivores had larger home ranges than terrestrial herbivores, though terrestrial mesocarnivores had the largest home ranges. We also found that aquatic herbivores had larger home ranges than both aquatic carnivores and aquatic mesocarnivores. Our results are important to consider for planning and management sectors, to avoid the implementation of ineffective conservation policies.

## Introduction

Home range, or the area in which an animal lives and moves on a periodic basis [1], has been a long-used concept in ecological studies. Several authors have reviewed methods for estimating home ranges [2,1,3] as well as trends in reporting home range estimations [4]. In comparison, using home ranges in planning or decision-making contexts is a relatively new idea [4]. However, there have not yet been any analyses reviewing home range studies in the context of trophic levels.

Trophic cascades can occur as a top-down process, where a change in the abundance, distribution, or behaviour of consumer species will trickle down the food chain to affect herbivores and vegetation or as a bottom-up process, where a change in vegetation will affect the abundance and distribution of herbivores and carnivores [5,6,7]. The same species can hold multiple trophic levels depending on the community composition of a region [8]. For example, a coyote in an area with wolves may be considered a mesocarnivore, or intermediate carnivore, and consume mainly hares, while a coyote in an area without wolves may be considered an apex carnivore and consume deer [7]. We could hypothesize that the coyote consuming mainly deer would have a larger home range than the coyote consuming mainly hares, because deer have larger habitat requirements than hares [7]. It would therefore be inaccurate to generalize a species’ home range estimate based on a single study, unless the authors made an explicit reference to the trophic level of the study species. Policy-makers typically use existing estimates of home range size for a species and generalise them across different geographic areas. This is problematic as home range size may change with trophic level; some species can occupy different trophic levels and this may lead to over or under estimating home ranges in a policy-based conservation approach.”

The objectives of this study were to 1) review the current relevant literature and critically analyse published articles with home range analyses, 2) to assess the most commonly-used methods of home range analysis by animal taxon, and 3) to compare home range size by species’ trophic level. This critical analysis will provide insight on current trends in home range studies and determine any significant differences in the home range size of species from different trophic levels. We hypothesize that if a species occupying a higher trophic level has larger habitat needs, it will then have a larger average home range. These are important considerations to improve recommended guidelines for conservation biology policies.

## Methods

This study involved surveying journal articles discussing animal home ranges from multiple journals, within a number of different animal species. Studies spanning a large geographic range were considered. A range of species classes were considered within this analysis, including mammals, birds, reptiles, amphibians, fish, and arthropods. The trophic cascade levels we considered were apex carnivore, mesocarnivore, and herbivore. For additional details on the study species or sites, please refer to the results or to the PRISMA checklist which can be found in S1 File.

### Literature search

We used the Institute for Scientific Information’s Web of Science database to search for articles containing the term ‘home range’ in the topic that were published between 2000 and 2015 to identify current trends in research articles. We chose to use articles that were cited a minimum of 25 times to base our study on widely-used literature. Research including primates as the study species were not included in this analysis due to their uniquely complex movement patterns [9].

To maintain the integrity of this analysis, we rejected all articles that did not report results from home range calculations. A future study taking into account additional complex movement patterns (such as migration) should include primates in its analyses. We also rejected articles that did not report sample size, though it should be noted that articles mentioning number of study animals and/or number of location points were included in this analysis. We then recorded details from each usable article based on 14 criteria listed in Table 1, including the year and journal of publication, the study species and location, and the software used in home range or site fidelity calculations. For additional details on reviewed literature, please refer to a complete list of articles used and their details, found in the S2 File.

**Table 1:** General details of study articles. We noted details from each article judged to be relevant based on the questions found in this table.

Year of publication
Journal
Study species
Taxonomy
Geographic location
Terrestrial or Aquatic
Site fidelity analyzed? (Y/N)
Number of animals studied
Number of location estimates
Method of home range analysis
Software used
Model created and/or used? (Y/N)
Number of species studied
Species trophic level
Discussed conservation (Y/N)

### Statistical analyses

We noted the average home range size from each relevant article. To control for study species size, we noted the mean body mass of the animals within the study. For example, an elk would have a significantly larger home range than a mouse, despite both being herbivores. If body mass was not reported in the article, we used various references to determine the mean mass of the species within the geographic range where the study took place, with field guides being our primary source. We compared average body masses reported in studies used in our review to average body masses cited in field guides. We used IBM SPSS to perform a paired T-test as well as Post Hoc analyses, and we found no significant differences. This suggests that the body masses cited in research articles were not significantly different from home ranges cited in range-specific field guides, and that using field guides to determine body mass for species in studies that did not report mass was an acceptable and accurate method for determining the average body mass of species.

We plotted home range against average body mass for each species in our review to determine whether a correlation was present. We plotted species both within a single trophic level and throughout all trophic levels. We measured the coefficient of determination (R^2^) for each plot using a best-fit trendline.

We controlled for mass with the equation:

$${\mathrm{HR}}_{\mathrm{ratio}}=\frac{\mathrm{average}\;\mathrm{home range}}{\mathrm{mean mass}}$$

where HR_ratio_ indicates the species’ average home range controlled for the species’ mean mass within the geographic range of the field study. We used these HR_ratio_ values in a one-way Analysis of Variance (ANOVA) to determine whether home range was correlated to trophic level. The three trophic levels we defined for this study (apex carnivore, mesocarnivore, and herbivore) were also tested against HR_ratio_ values in multiple post-hoc tests to determine where, if any, statistical significance was found. Terrestrial studies were analysed both separate and combined with aquatic studies. All statistical analyses were done using IBM SPSS v. 23.

## Results

### Literature results

**Fig 1:**
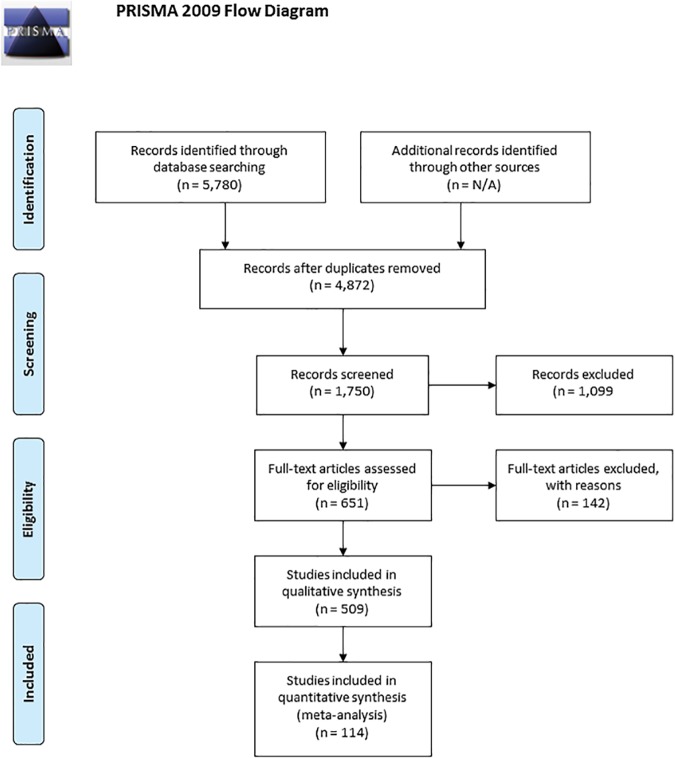
Results returned through literature searches in our meta-analysis.

The first search for home range on WOS resulted in 76,323 results, and we found 651 potentially relevant studies using the aforementioned criteria (Table 2). 142 of these studies were deemed irrelevant due to the main study species being a primate and were thus eliminated from our analysis. Of the 509 remaining studies, 395 did not report home range average or number of study animals or location points. The other 114 studies were used in our analysis (Fig 1).

**Table 2:** Summary of search results based on search terms, where quotation marks (“”) indicate a search for the exact term and an asterix (*) indicates all variations of the word.

Term	Number of results
Home range	76,323 results
“Home range”	27,833 results
Between 2000 and 2015	17,631 results
In North or South America	8,425 results
Animal*	5,780 results
Citation number ≥ 25	651 results (509 relevant)

The 114 articles we used were published between 2000 and 2012, with the most frequent publication year being 2002 (23 relevant articles). The articles used for our study covered 48 journals, with the three most recurrent journals being the Canadian Journal of Zoology, the Journal of Mammalogy, and the Journal of Wildlife Management, with 12, 10, and 8 relevant articles respectively. Study species ranged between 6 classes; most studies (71 of 114) were based on mammals, with a total of 108 mammalian home ranges reported. 84 articles were used in the terrestrial analyses, 29 results were used in the aquatic analyses, and 1 result was used in both (Table 3).

**Table 3:** Summary of article details. MCP refers to minimum convex polygons while KDE refers to Kernel density estimates. For more information on the reviewed literature and their details, please refer to complete list found in the Supplemental Information S2 File.

Criterion	Number of studies	Percentage of studies
Terrestrial	85	74.6
Aquatic	30	26.4
• Home range analysisMCP	59	51.8
• KDE	37	32.5
• Both	11	9.65
• Unspecified	7	6.14
Site fidelity calculated	36	31.6
Software reported	98	86.0
Model created	2	1.74
Multiple species studied	20	17.5
Direct species interaction in trophic cascade	5	4.39
Discussed conservation	45	39.5

The majority (59 of 114) of studies reported using minimum convex polygons (MCP) as their method of calculating home range, while 37 studies reported using Kernel density estimations (KDE), and an additional 11 studies reported using both MCP and KDE. 7 studies did not specify the method by which they calculated home ranges (Table 3). The majority of studies (91 of 114) reported the software used to calculate home ranges, though only 51 reported the software extension. 7 studies reported manually calculating home ranges without the use of software, and 16 studies did not specify whether a software was used or not (Table 3). Though many different software packages were used, the most frequent were the Animal Movements extension (ArcView), the CALHOME package (R), and the Home Ranger extension (ArcView) with 19, 14, and 12 respective reported uses. 2 studies created models to be used for their analyses (Table 3). 20 studies analysed more than one target species within the study area. Forty-five studies discussed applications concerning conservation (Table 3).

### Statistics results

**Fig 2:**
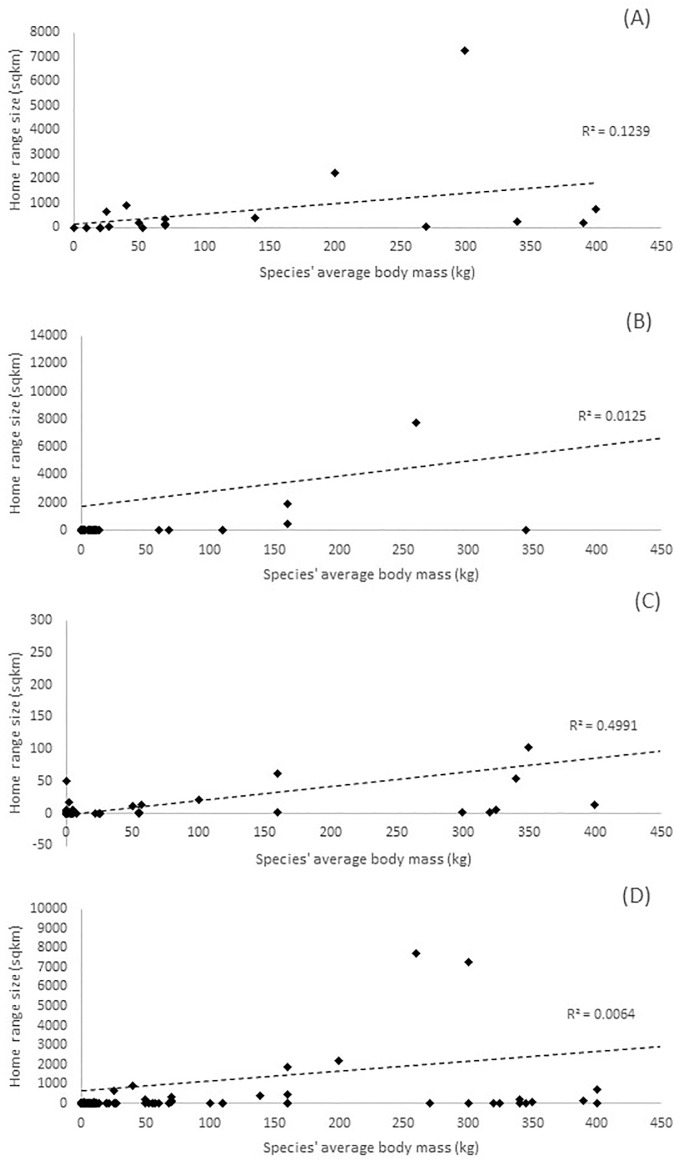
Species’ average body mass as compared to home range size. (A) represents all herbivorous species; (B) represents all mesocarnivorous species; (C) represents all apex carnivorous species; and (D) represents species across all trophic levels. The dashed lines represent best-fit linear trendlines with the coefficient of determination (R^2^).

We found a possible correlation between average body mass and home range size. This was most apparent in aquatic herbivores, where R^2^ = 0.8973. It was also apparent throughout herbivores in general, where R^2^ = 0.4991. There was a high amount of variation in these trends however, specially in terrestrial apex carnivores and herbivores (R^2^ = 0.1239 and R^2^ = 0.3545, respectively) as well as for mesocarnivores both terrestrial and aquatic (R^2^ = 0.1710 and R^2^ = 0.0006; Fig 2). Caution should thus be taken when assuming a direct correlation between body size and home range size.

**Fig 3:**
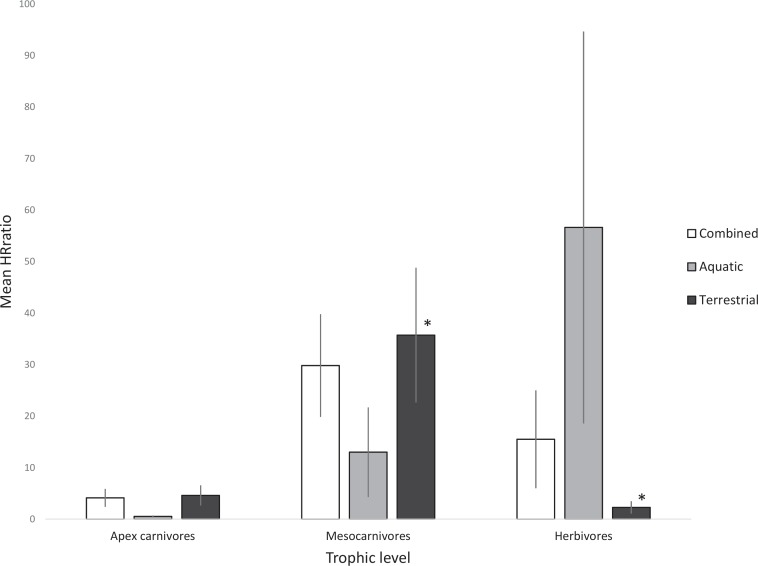
Mean HR_ratio_ by trophic level for each analysis. Asterisks indicate pairwise significant difference at p <0.05. Error bars illustrate standard error of each mean.

The analysis for terrestrial HR_ratio_ by trophic level returned statistically significant results when comparing herbivores and mesocarnivores (F_2,121_ = 4.110, *p* = 0.019). We also noticed a trend in analyses done for aquatic HR_ratio_ by trophic level, where apex carnivores showed smaller ranges than herbivores, though these results were not statistically significant. For combined terrestrial and aquatic HR_ratio_ by trophic level, mesocarnivores tended to have much larger home ranges than both herbivores and apex carnivores, though these results were statistically insignificant (Fig 3).

## Discussion

### Literature selection

Despite our careful criteria for article selection, we found a number of articles from a range of journals, published throughout several different years. The articles we chose also spanned a variety of taxa in multiple different countries. Though we would likely have discovered additional articles using broader search criteria, the studies we used included enough relevant literature to find trends in home ranges. It should be noted that it is generally difficult to compare results from different home range studies as each study species varies in spatial distribution and habitat requirements [4]; defining trophic level at the population scale, rather than on a species-wide scale, as well as controlling for species’ body mass allowed us to overcome this issue and compare results from multiple studies.

Of the 651 articles returned in our initial search, only 114 articles were suitable for our analysis. Many studies did not report specific home range estimates, although all relevant articles analysed and discussed home range in some capacity. A relatively recent review of home range analyses by Laver and Kelly (2008) reported that many authors did not include more recent advances in home range estimations, and that many authors did not report the software extensions or versions they used. We did not find this to be a widespread issue with only 16 articles not specifying the software used in their home range estimations. The greatest restriction we found in our selection of articles was that many authors did not publish the number of study animals or location data points. The omission of any of these details could affect experiment reproducibility and credibility [2,1,4]. Increasing reporting standards in ecological papers, such as number of GPS fixes or sample size, could remedy this issue.

### Study details

The majority of articles that qualified for analysis based on our criteria studied species that were primarily terrestrial. We found articles that spanned 6 classes (*mammalia*, *aves*, *reptilian*, *amphibia*, *and pisces*). This variety of taxonomic groups indicates that our search criteria led to a reasonable overview of the current and relevant literature. Approximately half of all studies used only minimum convex polygons (MCP) to estimate home range. Several authors using MCP chose to increase accuracy through excluding a percentage of outlier locations and analysing core area MCP. Alternatively, multiple studies used both MCP and Kernel density estimations (KDE) to gauge home range to increase overall accuracy. Only two studies involved creating and using a movement model. Both models were created based on ecologic location data, with a study-specific purpose. This specificity greatly increases the accuracy of their resulting outcomes [10,11].

The majority of articles that qualified for our study only analysed a single species, though some analysed multiple species which interact indirectly, through apparent competition or niche partitioning. Studies involving a more comprehensive overview of the environment [12] may result in estimates which are likely to be much closer reflections of actual species’ movement [13]. The majority of articles only analysed a single target species independent from other species within its food web, with some of these recommending implementation of their results in a conservation context. Any guidelines or policies occurring from the implementation of these recommendations may consequently be ineffective as interactions between species can alter home range requirements [13,12]. Guideline and policy recommendations should ideally emerge from studies including multiple species’ interactions, as the interactions between species often alters species’ movement patterns [12].

Our rigorous standards for data reporting for articles led to a fairly small sample size for our review. As a result, most of our multivariate analyses did not yield significant results. We discuss patterns found throughout our study rather than draw concrete conclusions from our admittedly limited results.

### Implications of trophic levels in policy-based conservation

The typical “10 percent conservation goal” was appropriated by Miller (1984)[14] from a study by Myers (1979)[15] suggesting that 10 to 20 percent of moist forest habitat would need to be conserved to sustain the ecosystem services humans require [14,16]. Unfortunately, this “10 percent conservation goal” has been further appropriated by a wide number of policy-makers, despite being an arbitrary and, in the case of most species, ecologically insufficient quantity of habitat [16]. This example illustrates the potential detriment of poor conservation planning that we can aim to avoid in the future.

Less than 20% of all studies that qualified for our analyses considered trophic level, though almost 40% of studies made recommendations for improving conservation policies. This oversight could be detrimental when translating results into policies. We found that sedentary herbivores tend to have fairly stable ranges, and they would likely benefit more from protected areas. This is especially true for ecologically resilient herbivores, such as elk or deer, in constrast with ecologically sensitive species such as boreal woodland caribou, or for migratory herbivores, such as manatees [17]. We also found that apex predators tend to have larger and typically more nomadic ranges [18]; this can be attributed to their large habitat requirements for territory, mate acquisition, and foraging [18]. Our results suggest that conservation policies, especially those generalised from other study species, such as the Miller (1984) & Myers (1979) case, would offer inadequate habitat for apex carnivores. Moreover, top predators tend to be more ecologically sensitive especially towards anthropogenic disturbance [18]. Following the minimal protected area conservation requirement without allowing for a natural buffer zone would further decrease the available habitat for these species [19]. We found that mesocarnivores tended to have the largest range size of all the trophic levels; this can be attributed to inter-specific competition for forage between mesocarnivores and apex carnivores [7]. For example, wolves may challenge a coyote for access to a carcass and displace it, which results in an expanded foraging range for the coyote [7]. Another contributor to mesocarnivore range size could be their comparative resiliency; many terrestrial mesocarnivore species (e.g. raccoons, coyotes, foxes) can adapt to live in close proximity to human infrastructure [7]. Mesocarnivores tend to do well around human development because they are adaptable and behaviourally plastic [7,19]. Given this, they would be less affected by a conservation strategy that attempts to define a protected area within a landscape characterized by human development. However, despite their resiliency, it is damaging to the entire ecosystem to generalise conservation strategies that are specific to a single species or that overlook essential ecological aspects, such as trophic level.

### Correlation between body mass and home range sizes

While we found general trends between average body size and home range, we also noticed a great amount of variation in species. In terrestrial species, the trends were more noticeable. This is likely due to the relatively small number of migratory terrestrial species in our review. Though we did not specifically select articles without migratory terrestrial species, many articles studying such species did not conform to our rigorous article selection standards. Comparatively, a much greater proportion of aquatic studies in our review contained migratory species, which may have skewed the results of our body mass to home range correlation analysis. Additionally, a large number of studies with bats as the species of interest were included in our review. Bats have a relatively small body mass but relatively large home ranges. It is likely that this also skewed our analyses. We suggest that researchers using multiple species of interest with significantly different average body masses may assume that larger species will have larger home ranges, but should use caution when drawing conclusions about home range size based solely on average body mass.

### Exclusion of environmental productivity and subsequent limitations

While our analysis does not account for environmental productivity, it does account for the geographic range of studied species. Still, omitting environmental productivity has the possibility to influence our results as environmental productivity would undoubtedly have an impact on average home range size [20]. Animals within an ecosystem with lower productivity would likely have a much larger home range than animals of the same species within an ecosystem with higher productivity, as the habitat requirements to obtain adequate forage in an ecosystem with lower productivity would likely be greater [20]. While body size could be used as a proxy for environmental productivity [21], there are a number of unrelated ecological factors that could influence body size (see Bergmann’s rule; 22,23,24]. As a result of the ambiguous nature of body size as a measure of productivity and the lack of specification of ecozones in which studies took place, we did not control for environmental productivity in our study.

### Conclusion

The standards of reporting details of home range studies should be increased to improve reproducibility and credibility of spatial ecological studies. Many studies used either minimum convex polygons or Kernel density estimations to determine home range estimates which can lead to inaccurate results, though a few authors chose to use both methods to improve the accuracy of their estimations. Reporting sample size of study animals and/or of location points in home range estimates would also greatly increase the reproducibility of published studies. Several articles studying a single target species made recommendations from implementation to improve conservation guidelines and policies; this could lead to ineffective conservation as actual species’ movement would likely differ greatly from modelled species’ movement. The risk of implementing inefficient policies greatly increases with the oversight of trophic level and multiple species’ interactions. This could lead to ineffective guidelines and regulations that would have no ecological benefit–or be a detriment in themselves–to conservation species. A future review incorporating a greater number of studies would be beneficial to supplement the trends and patterns noticed throughout this review. Less rigorous reporting standards would allow for a larger range of studies to be analysed in the future. Future guideline or policy implementations would also greatly benefit from studies with multiple target species in order to ensure effective planning for conservation.

## Supporting information

S1 File PRISMA checklist. The following checklist reports information found in our review.
(DOCX)

S2 File Articles used in our review. The following articles conformed to our rigorous article selection standards and thus were analysed for our review.
(DOCX)
