# Supplementary material for: A meta-analysis of home range studies in the context of trophic levels: Implications for policy-based conservation
Source: PLoS One. 2017 Mar 7;12(3):e0173361. doi: 10.1371/journal.pone.0173361 (PMC5340398; doi:10.1371/journal.pone.0173361)
Supplement: S2 File — The following articles conformed to our rigorous article selection standards and thus were analysed for our review. (DOCX) [file pone.0173361.s002.docx]

**SUPPLEMENTAL INFORMATION 1 Articles used in our review**

The following articles conformed to our rigorous article selection standards and thus were analysed for our review.

Amstrup, S.C., Durner, G.M, Stirling, I., Lunn, N.J., and Messier, F. 2000. Movements and distribution of polar bears in the Beaufort Sea. Canadian Journal of Zoology 78: 948-966

Anderson, D.P., Forester, J.D., Turner, M.G., Frair, J.L, Merrill, E.H., Fortin, D., Mao, J.S., and Boyce, M.S. 2005. Factors influencing female home range sizes in elk (*Cervus elaphus*) in North American Landscapes. Landscape Ecology 20: 257-271

Anich, N.M., Benson, T.J., and Bednarz, J.C. 2009. Estimating territory and home-range sizes: Do singing locations alone provide an accurate estimate of space use? The Auk 126(3): 626-634

Arvisais, M., Bourgeois, J.C., Lévesque, E., Daigle, C., Masse, D., and Jutras, J. 2002. Home range and movements of a wood turtle (*Clemmys insculpta*) population at the northern limit of its range. Canadian Journal of Zoology 80:402-408

Austin, D., Bowen, W.D., and McMillan, J.I. 2004. Intraspecific variation in movement patterns: Modeling individual behaviour in a large marine predator. Oikos 105(1): 15-30

Beasley, J.C., Devault JR, T.L., and Rhodes, O.E. 2007. Home range attributes of raccoons in a fragmented agricultural region of northern Indiana. Journal of Wildlife Management 71(3): 844-850

Beckmann, J.P., and Berger, J. 2003. Using black bears to test ideal-free distribution models experimentally. Journal of Mammalogy 84(2): 594-606

Berger, K.M., and Gese, E.M. 2007. Does interference competition with wolves limit the distribution and abundance of coyotes? Journal of Animal Ecology 76(6): 1075-1085

Bernard, E., and Fenton, M.B. 2003. Bat mobility and roosts in a fragmented landscape in Central Amazonia, Brazil. Biotropica 35(2): 262-277

Bixler, A., and Gittleman, J.L. 2000. Variation in home range and use of habitat in the striped skunk (*Mephitis mephitis*). Journal of Zoology 251(4): 525-533

Blundell, G.M., Ben-David, M., and Bowyer, R.T. 2002. Sociality in river otters: cooperative foraging or reproductive strategy. Behavioural Ecology 13(1): 134-141

Bontadina, F., Schofield, H., and Naef-Daenzer, B. 2002. Radio-tracking reveals that lesser horseshoe bats (*Rhinolophus hipposideros*) forage in woodland. Journal of Zoology 258(3): 281-290

Bowyer, R.T., and Kie, J.G. 2006. Effets of scale on interpreting life-history characteristics of ungulates and carnivores. Diversity and Distributions 12: 244-257

Breed, G.A., Bowen, W.D., McMillan, J.I., and Leonard, M.L. 2006. Sexual segregation of seasonal foraging habitats in a non-migratory marine mammal. Biological Sciences 273(1599): 2319-2326

Breen, M.J., Ruetz,C.R., Thompson, K.J., an Kohler, S.L. 2009. Movements of mottled sculpins (*Cottus bairdii*) in a Michigan stream: how restricted are they? Canadian Journal of Fisheries and Aquatic Sciences 66: 31-41

Burrell, K.H., Isely, J.J., Bunnell, D.B., Van Lear, D.H., and Dolloff, C.A. 2000. Seasonal movement of brown trout in a southern Appalachian river. Transactions of the American Fisheries Society 129(6): 1373-1379

Cáceres, N.C., and Monteiro-Filho, L.A. 2001. Food habits, home range and activity of *Didelphis aurita* (Mammalia, Marsupialia) in a forest fragment of southern Brazil. Studies on Neotropical Fauna and Environment 36(2): 85-92

Capaldi, E.A., Smith, A.D., Osborne, J.L., Fahrbach, S.E., Farris, S.M., Reynolds, D.R., Edwards, A.S., Martin, A., Robinson, G.E., Poppy, G.M., and Riley, J.R. 2000. Ontogeny of orientation flight in the honeybee revealed by harmonic radar. Nature 403: 537-540

Carlson, J.K., Heupel, M. R., Bethea, D.M., and Hollensead, L.D. 2008. Coastal habitat use and residency of juvenile Atlantic sharpnose sharks (*Rhizoprionodon terraenovae*). Estuaries and Coasts 31(5): 931-940

Chapman, M.R., and Kramer, D.L. 2000. Movements of fishes within and among fringing coral reefs in Barbados. Environmental Biology of Fishes 57: 11-24

Ciarniello, L.M., Boyce, M.S., Seip, D.R., and Heard, D.C. 2007. Grizzly bear habitat is scale dependant. Ecological Applications 17(5): 1424-1440

Collins, A.B., Heupel, M.R., and Motta, P.J. 2007. Residence and movement patterns of cownose rays *Rhinoptera bonasus* within a south-west Florida estuary. Journal of Fish Biology 71: 1159-1178

Cote, D., Moulton, S., Frampton, P.C.B., Scruton, D.A., and McKinley, R.S. 2004. Habitat use and early winter movements by juvenile Atlantic cod in a coastal area in Newfoundland. Journal of Fish Biology 64: 665-679

Cotton, C.L., and Parker, K.L. 2000. Winter habitat and nest trees used by Northern flying squirrels in subboreal forests. Journal of Mammalogy 81(4): 1071-1086

Covert-Bratland, K.A., Block, W.M., and Theimer, T.C. 2006. Hairy woodpecker winter ecology in Ponderosa pine forests representing different ages since wildfires. Journal of Wildlife Management 70(5): 1379-1392

Cramer, P.C., and Portier, K.M. 2001. Modeling Floride panther movements in response to human attributes of the landscape and ecological settings. Ecological Modelling 140: 51-80

Crook, D.A. 2004. Is the home range concept compatible with the movements of two species of lowland river fish? Journal of Animal Ecology 73: 353-366

Crooks, R. 2002. Relative sensitivities of mammalian carnivores to habitat fragmentation. Conservation Biology 16(2): 488-502

Cutrera, A.P., Antinuchi, C.D., Mora, M.S., and Vassallo, A.I. 2006. Home range and activity patterns of the South American subterranean rodent *Ctenomys talarum*. Journal of Mammalogy 87(6): 1183-1191

Dahle, B., and Swenson, J.E. 2003. Seasonal range size in relation to reproductive strategies in brown bears *Ursus arctos*. Journal of Animal Ecology 72: 660-667

Deutsch, C.J., Reid, J.P., Bonde, R.K., Easton, D.E., Kochman, H.I., and O’Shea, T.J. 2003. Seasonal movements, migratory behaviour, and site fidelity of West Indian manatees along the Atlantic coast of the United States. Wildlife Monographs 151: 1-77

Dickson, B.G., and Beier, P. 2002. Home-range and habitat selection by adult cougars in Southern California. Journal of Wildlife Management 66(4): 1235-1245

Dillon, A., and Kelly, M.J. 2008. Ocelot home range, overlap and density: comparing radio telemetry with camera trapping. Journal of Zoology 275: 391-398

Dussault, C., Ouellet, J.P., Courtois, R., Huot, J., Breton, J., and Jolicoeur, H. 2005. Linking moose habitat selection to limiting factors. Ecography 28: 619-628

Eide, N. E., Jepsen, J.U., and Prestrud, P. 2004. Spatial organisation of reproductive arctic foxes *Alopex lagopus*: Responses to change in spatial and temporal availability of prey. Journal of Animal Ecology 73(6): 1056-1068

Elchuk, C.L., and Wiebe, K.L. 2003. Home range size of northern flickers (*Colaptes auratus*) in relation to habitat and parental attributes. Canadian Journal of Zoology 81: 954-961

Etter, D.R., Hollis, K.M., Van Deelen, T.R., Ludwig, D.R., Chelsvig, J.E., Anchor, C.L., and Warner, R.E. 2002. Survival and movements of white-tailed deer in suburban Chicago, Illinois. Journal of Wildlife Management 66(2): 500-510

Foerster, C.R., and Vaughan, C. 2002. Home range, habitat use, and activity of the Baird’s Tapir in Costa Rica. Biotropica 34(3): 423-437

Forester, J.D., Ives, A.R., Turner, M.G., Anderson, D.P., Fortin, D., Beyer, H.L., Smith, D.W., and Boyce, M.S. 2007. State-space models link elk movement patterns to landscape characteristics in Yellowstone National Park. Ecological Monographs 77(2): 285-299

Gehrt, S.D., Anchor, C., and White, L.A. 2009. Home range and landscape use of coyotes in a metropolitan landscape: Conflict of coexistence? Journal of Mammalogy 90(5): 1045-1057

Gilchrist, J.S., and Otali, E. 2002. The effects of refuse-feeding on home range use, group size, and intergroup encounters in the banded mongoose. Canadian Journal of Zoology 80: 1795-1802

Gosselink, T.E., Van Deelen, T.R., Warner, R.E., and Joselyn, M.G. 2003. Temporal habitat partitioning and spatial use of coyotes and red foxes in Est-Central Illinois. Journal of Wildlife Management 67(1): 90-103

Greenleaf, S.S., Williams, N.M., Winfree, R., and Kremen, C. 2007. Bee foraging ranges and their relationship to body size. Oecologia 153(3): 589-596

Grigione, M.M., Beier, P., Hopkins, R.A., Neal, D., Padley, W.D., Schonewald, C.M., and Johnson, M.L. 2002. Ecological and allometric determinants of home-range size for mountain lions (*Puma concolor*). Animal Conservation 5: 317-324

Grinder, M.I., and Krausman, P. R. 2001. Home range, habitat use, and nocturnal activity of coyotes in an urban environment. Journal of Wildlife Management 65(4): 887-898

Gubbins, C. 2002. Use of home ranges by resident bottlenose dolphins (*Tursiops truncates*) in a South Carolina Estuary. Journal of Mammalogy 83(1): 178-187

Haenel, G.J., Smith, L.C., and John-Alder, H.B. 2003. Home-range analysis in *Sceloporus undulates* (Eastern fence lizard): Spacing patterns and the context of territorial behavioural. Copeia 1: 99-112

Hamer, T.E., Forsman, E.D., Glenn, E.M. 2007. Home range attributes and habitat selection of barred owls and spotted owls in an area of sympatry. The Condor 109(4): 750-768

Hanski, I.K., Stevens, P.C., Ihalempiä, P., and Selonen, V. 2000. Home range size, movements, and nest-site use in the Siberian flying squirrel *Pteromys Volans*. Journal of Mammalogy 81(3): 798-809

Hawkes, L.A., Witt, M.J., Broderick, A.C., Coker, J.W., Coyne, M.S., Dodd, M., Frick, M.G., Godfrey, M.H., Griffin, D.B., Murphy, S.R., Murphy, T.M., Williams, K.L., and Godley, B.J. 2011. Home on the range: spatial ecology of loggerhead turtles in Atlantic waters of the USA. Diversity and Distributions 17: 624-640

Haywarth, M.W., de Tores, P.J., Augee, M.L., Fox, B.J., and Banks, P.B. 2004. Home range and movements of the quokka *Setonix brachyurus* (Macropodidae: Marsupialia), and its impact on the viability of the metapopulation on the Australian mainland. Journal of Zoology 263: 219-228

Heide-Jorgensen, M.P., Dietz, R., Laidre, K.L., and Richard. P. 2002. Autumn movements, home ranges, and winter density of narwhals (*Monodon momoceros*) tagged in Tremblay Sound, Baffin Island. Polar Biology 25: 331-341

Henry, M., Thomas, D.W., Vaudry, R., and Carrier, M. 2002. Foraging distances and home range of pregnant and lactating little brown bats (*Myotis lucifugus*). Journal of Mammalogy 83(3): 767-774

Herfindal, I., Linnell, J.D.C., Odden, J., Nilsen, E.B., and Anderson, R. 2005. Prey density, environmental productivity and home range size in the Eurasian lynx (*Lynx lynx).* Journal of Zoology 265(1): 63-71

Heupel, M. R., Simpfendorfer, C.A., and Heuter, R.E. 2003. Estimation of shark home ranges using passive monitoring techniques. Environmental Biology of Fishes 71: 135-142

Heupel, M.R., Simpfendorfer, C.A., Collins, A.B., and Tyminski, J.P. 2006. Residency and movement patterns of bonnethead sharks *Sphyrna tiburo* in a large Florida estuary. Environmental Biology of Fishes 76: 47-67

Hingrat, Y., Jalme, M.S., Ysnel, F., Lacroix, F., Seabury, J., and Rautureau, P. 2004. Relationships between home range size, sex and season with reference to the mating system of the Houbara Bustard *Chalmydotis undulata undulata*. Ibis 146: 314-322

Jadot, C., Donnay, A., Acolas, M.L., Cornet, Y., and Bégout Anras, M.L. 2006. Activity patterns, home range size, and habitat utilization of *Sarpa salpa* (Teleostei: Sparidae) in the Mediterranean Sea. Journal of Marine Sciences 63: 128-139

Johnston, B., and Frid, L. 2002. Clearcut logging restricts the movements of terrestrial Pacific giant salamanders (*Dicamptodon tenebrosus* Good). Canadian Journal of Zoology 80(12): 2170-2177

Kelly, B.P., Badajos, O.H., Kunnasranta, M., Moran, J.R., Marinez-Bakker, M., Wartzok, D., and Boveng, P. 2010. Seasonal home ranges and fidelity to breeding sites among ringed seals. Polar Biology 33:1095-1109

Kie, J.G., Bowyer, R.T., Nicholson, M.C., Boroski, B.B., and Loft, E.R. 2002. Landscape heterogeneity at different scales: Effects on spatial distribution of mule deer. Ecology 83(2):530-544

Kilpatrick, H.J., Spohr, S., and Lima, K.K. 2001. Effets of population reduction on home ranges of female white-tailed deer at high densities. Canadian Journal of Zoology 79: 949-954

King, S.R.B. 2002. Home range and habitat use of free-ranging Przewalski horses at Hustai National Park, Mongolia. Applied Animal Behaviour Science 78:103-113

Kjellander, P., Hewinson, A.J.M, Liberg, O., Angibault, J.M., Bideau, E., and Cargnelutti, B. 2004. Experimental evidence for density-dependence of home range size in roe deer (*Capreolus capreolus L*). Oecologia 3:478-485

Kwiatkowski, A., and Sullivan, B.K. 2002. Geographic variation in sexual selection among populations of an Iguanid lizard, *Sauromalus ater*. Evolution 56(10): 2039-2051

Laurian, C., Dussault, C., Ouellet, J.P., Courtois, R., Poulin, M., and Breton, L. 2008. Behaviour of moose relative to road network. Journal of Wildlife Management 72(7): 1550-1557

Lesage, L., Crête, M., Huot, J., Dumont, A., and Ouellet, J.P. 2000. Seasonal home range size and philopatry in northern white-tailed deer populations. Canadian Journal of Zoology 78(11):1930-1940

Litzgus, J. D., and Mousseau, T.A. 2004. Home range and seasonal activity of southern spotted turtles (*Clemmys guttata*): Implications for management. Copeia 2004(4):804-817

Lurz, P.W.W., Garson, P.J., and Wauters, L.A. 2000. Effects of temporal and spatial variations in food supply on the space and habitat use of red squirrels (*Sciurus vulgaris L*.). Journal of Zoology 251(2): 167-178

Mabry, K.E., and Barrett, G.W. 2002. Effects of corridors on home range sizes and interpatch movements of three small mammal species. Landscape Ecology 17: 629-636

Maehr, D.S., Land, E.D., Shindle, D.B., Bass, O.L., and Hoctor, T.S. 2002. Florida panther dispersal and conservation. Biological Conservation 106:187-197

Makarieva, A.M., Gorshkov, V.G., and Li, B.L. 2005. Why do population density and inverse home range scale differently with body size? Implications for ecosystem stability. Ecological Complexity 2:259-271

Makowski, C., Seminoff, J.A., and Salmon, M. 2006. Home range and habitat use of juvenile Atlantic green turtkes (*Chelonia mydas L.*) on shallow reef habitats in Palm Beach, Florida, USA. Marine Biology 148:1167-1179

Marshell, A., Mills, J.S., Rhodes, K.L., and McIlwain, J. 2011. Passive acoustic telemetry reveals highly variable home range and movement patterns among unicornfish within a marine reserve. Coral Reefs 30:631-642

Marzluff, J.M., and Neatherlin, E. 2006. Corvid response to human settlements and campgrounds: Causes, consequences, and challenges for conservation. Biological Conservation 130:301-314

Marzluff, J.M., Millspaugh, J.J., Hurvitz, P., and Handcock, M.S. 2004. Relating resources to a problematic measure of space use: Forest fragments and Steller’s jays. Ecology 85(5):1411-1427

Mazzotti, S., Pisapia, A., and Fasola, M. 2002. Activity and home range of *Testudo hermanni* in Northern Italy. Amphibia-Reptilia 22: 305-312

McLellan, B.N., and Hovey, F.W. 2001. Natal dispersal of grizzly bears. Canadian Journal of Zoology 79(5):838-844

McLoughlin, P.D., Case, R.L., Gau, R.J., Cluff, H.D., Mulders, R., and Messier, F. 2002. Hierarchical habitat selection by barren-ground grizzly bears in the central Canadian Arctic. Oecologia 132(1):102-108

McLoughlin, P.D., Ferguson, S.H., and Messier, F. 2000. Intraspecific variation in home range overlap with habitat quality: a comparison among brown bear populations. Evolutionary Ecology 14:39-60

Menzel, J.M., Ford, W.M., Menzel, M.A., Carter, T.C., Gardner, J.E., Garner, J.D., and Hofmann, J.E. 2005. Summer habitat use and home range analysis of the endangered Indiana bat. Journal of Wildlife Management 69(1):430-436

Menzel, M.A., Menzel, J.M., Ford, W.M., Edwards, J.W., Carter, T.C., Churchill, J.B., and Kilgo, J.C. 2001. Home range and habitat use of male Rafinesque’s big-eared bats (*Corynorhinus rafinesquii*). The American Midland Naturalist 145(2):402-408

Meyer, C.F.J., Weinbeer, M., and Kalko, E.K.V. 2005. Home-range size and spacing patterns of *Macrophyllum macrophyllum* (Phyllostomidae) foraging over water. American Society of Mammalogists 86(3):587-598

Meyer, C.G., and Holland, K.N. 2005. Movement patterns, home range size and habitat utilization of the bluespine unicornfish, *Naso unicornis* (Acanthuridae) in a Hawaiian marine reserve. Environmental Biology of Fishes 73:201-210

Meyer, C.G., Holland, K.N., Wetherbee, B.M., and Lowe, C.G. 2000. Movement patterns, habitat utilization, home range size and site fidelity of the whitesaddle goatfish, *Parupeneus porphyreus*, in a marine reserve. Environmental Biology of Fishes 59: 235-242

Mills, K.J., Patterson, B.R., and Murray, D.L. 2006. Effects of variable sampling frequencies on GPS transmitter efficiency and estimated wolf home range size and movement distance. Wildlife Society Bulletin 34(5):1463-1469

Mitchell, M.S., and Powell, R.A. 2007. Optimal use of resources structures home ranges and spatial distribution of black bears. Animal Behaviour 74:219-230

Moore, J.A., and Gillingham, J.C. 2006. Spatial ecology and multi-scale habitat selection by a threatened rattlesnake: The Eastern Massassauga (*Sistrurus catenatus catenatus*). Copeia 2006(4): 742-751

Morzillo, A.T., Feldhamer, G.A., and Nicholson, M.C. 2003. Home range and nest use of the golden mouse (*Ochrotomys nuttalli*) in Southern Illinois. Journal of Mammalogy 84(2):553-560

Mosnier, A., Ouellet, J.P., Sirois, L., and Fournier, N. 2003. Habitat selection and home range dynamics of the Gaspé caribou: a hierarchical analysis. Canadian Journal of Zoology 81: 1174-1184

Muths, E. 2003. Home range and movements of Boreal toads in undisturbed habitat. Copeia 20003:160-165

Mysterud, A., Pérez-Barbería, F.J., and Gordon, I.J. 2001. The effect of season, sex, and feeding style on home range area versus body mass scaling in temperate ruminants. Oecologia 127(1): 30-39

Nupp, T.E., and Swihart, R.K. 2000. Landscape-level correlates of small mammal assemblages in forest fragments of farmland. Journal of Mammalogy 81(2):512-526

Osterwalder, K., Klingenbock, A., and Shine, R. 2004. Field studies on a social lizard: Home range and social organization in an Australian skink, *Egernia major*. Austral Ecology 29: 241-249

Ovidio, M., Philippart, J.C., and Baras, É. 2000. Methodological bias in home range and mobility estimates when locating radio-tagged trout, *Salmo trutta*, at different time intervals. Aquatic Living Resources 13: 449-454

Owen, S.F., Menzel, M.A., Ford, W.M., Chapman, B.R., Miller, K.V., Edwards, J.W., and Wood, P.B. 2003. Home range size and habitat used by the Northern Myotis (*Myotis septentrionalis*). The American Midland Naturalist 150(2): 352-359

Plummer, M.V., and Mills, N.E. 2000. Spatial ecology and survivorship of resident and translocated hognose snakes (*Heterodon platirhinos*). Journal of Herpetology 34(4): 565-575

Prange, S., Gehrt, S.D., and Wiggers, E.P. 2004. Influences of anthropogenic resources on raccoon (*Procyon lotor*) movements and spatial distribution. Journal of Mammalogy 85(3): 483-490

Rechinsky, E.L., and Wetherbee, B.M. 2003. Short-term movements of juvenile and neonate sandbar sharks, *Carcharhinus plumbeus*, on their nursery grounds in Delaware Bay. Environmental Biology of Fishes 68: 113-128

Rettie, W.J., and Messier, F. 2001. Range use and movement rates of woodland caribou in Saskatchewan. Canadian Journal of Zoology 79(11): 1933-1940

Riley, S.P.D., Sauvajot, R.M., Fuller, T.K., York, E.C., Kamradt, D.A., Bromley, C., and Wayne, R.K. 2003. Effects of urbanization and habitat fragmentation on bobcats and coyotes in Southern California. Conservation Biology 17(2): 566-576

Robson, B.W., Goebel, M.E., Baker, J.D., Ream, R.R., Loughlin, T.R., Francis, R.C., Antonelis, G.A., and Costa, D.P. 2004. Separation of foraging habitat among breeding sites of a colonial marine predator, the northern fur seal (*Callorhinus ursinus*). Canadian Journal of Zoology 82: 20-29

Row, J.R., and Blouin-Demers, G. 2006. Kernels are not accurate estimators of home range size for herpetofauna. Copeia 2006(4): 797-802

Russo, D., Jones, G., and Migliozzi, A. 2001. Habitat selection by the Mediterranean horseshoe bat, *Rhinolophus Euryale* (Chiroptera: Rhinolophidae) in a rural area of southern Italy and implications for conservation. Biological Conservation 107: 71-81

Saïd, S., and Servanty, S. 2005. The influence of landscape structure on female roe deer home range size. Landscape Ecology 20: 1003-1012

Saïd, S., Gaillard, J.M., Duncan, P., Guillon, N., Guillon, N., Servanty, S., Pellerin, M., Lefeuvre, K., Martin, C., and Van Laere, G. 2005. Ecological correlates of home range size in spring-summer for female roe deer (*Capreolus capreolus*) in a deciduous woodland. Journal of Zoology 267(3): 301-308

Schofield, G., Hobson, V.J., Fossette, S., Lilley, M.K.S., Katselidis, K.A., and Hays, G.C. 2010. Fidelity to foraging sites, consistency of migration routes and habitat modulation of home range by sea turtles. Diversity and Distributions 16: 840-853

Semlitsch, R.D., and Bodie, J.R. 2003. Biological criteria for buffer zwnes around wetlands and riparian habitats for amphibians and reptiles. Conservation Biology 17(5): 1219-1228

Silvius, K.M., and Fragoso, J.M.V. 2003. Red-rumped agouti (*Dasyprocta leporine*) home range use in an Amazonian forest: Implications for the aggregated distribution of forest trees. Biotropica 35(1): 74-83

Singer, F.J., Zeigenfuss, L.C., and Spicer, L. 2001. Role of patch size, disease, and movement in rapid extinction of bighorn sheep. Conservation Biology 15(5): 1347-1354

Smith, A.C., and Schaefer, J.A. 2002. Home range size and habitat selection by American marten (*Martes americana*) in Labrador. Canadian Journal of Zoology 80: 1602-1609

Stone, P.A., and Baird, T.A. 2002. Estimating lizard home range: The Rose model revisited. Journal of Herpetology 36(3): 427-436

Waldron, J.L., Bennett, S.H., Welch, S.M., Dorcas, M.E., Lanham, J.D., and Kalinowsky, W. 2006. Habitat specificity and home range size as attributes of species vulnerability to extinction: a case study using sympatric rattlesnakes. Animal Conservation 9: 414-420

Welsh, J.Q., and Bellwood, D.R. 2012. Spatial ecology of the steephead parrotfish (*Chlorurus microrhinos*): an evaluation using acoustic telemetry. Coral Reefs 31: 55-65

Yasuda, T., and Arai, N. 2005. Fine-scale tracking of marine turtles using GPS-Argos PTTs. Biological Science 22: 547-553
